# Metabolic dysfunction-associated steatotic liver disease in people living with HIV: mechanisms, diagnosis, and management

**DOI:** 10.3389/fimmu.2026.1875819

**Published:** 2026-09-16

**Authors:** Ruoli Yu, Wei Xu, Jun Chen

**Affiliations:** Shanghai Public Health Clinical Center, Fudan University, Shanghai, China

**Keywords:** chronic inflammation, gut–liver axis, HIV, immune dysregulation, MASLD

## Abstract

Metabolic dysfunction-associated steatotic liver disease (MASLD) is the most common chronic liver disease worldwide and is increasingly recognized as a major contributor to liver-related and cardiometabolic morbidity. In people living with HIV (PLWH), the burden of MASLD is disproportionately high and continues to rise in the era of effective antiretroviral therapy (ART). The recent redefinition of MASLD underscores a paradigm shift toward metabolic dysfunction as a central driver of disease pathogenesis, a concept that is particularly relevant in the context of HIV infection. In PLWH, MASLD may represent a distinct immunometabolic condition in which conventional metabolic risk factors interact with chronic immune activation, persistent inflammation, ART exposure, and gut–liver axis dysfunction. These factors may contribute to steatosis and fibrosis and complicate conventional obesity-based screening and risk stratification. This narrative review summarizes the disease burden, immunometabolic mechanisms, diagnosis, and management of MASLD in PLWH and identifies priorities for research and clinical care. Management requires integrated assessment of metabolic dysfunction, persistent inflammation, cumulative ART exposure, and cardiovascular risk rather than reliance on obesity-based screening alone.

## 1 Introduction

The widespread use of antiretroviral therapy (ART) has substantially improved the survival of PLWH, shifting the clinical burden of HIV infection from AIDS-defining illnesses to chronic non-AIDS comorbidities. Among these, chronic liver disease has emerged as a leading cause of morbidity and mortality in this population (1).

Metabolic dysfunction-associated steatotic liver disease (MASLD), formerly termed nonalcoholic fatty liver disease (NAFLD), is increasingly recognized as a prevalent and clinically significant liver condition in PLWH (2). MASLD encompasses a pathological spectrum ranging from simple steatosis to metabolic dysfunction-associated steatohepatitis (MASH), which may progress to advanced fibrosis, cirrhosis, and hepatocellular carcinoma (HCC) (3). In PLWH, the burden of NAFLD is rising in parallel with increasing life expectancy, and is associated with adverse hepatic and extrahepatic outcomes, including cardiometabolic complications and reduced quality of life (4, 5). Throughout this review, MASLD and MASH are used when referring to the current nomenclature. When discussing studies published under the earlier NAFLD/NASH framework, we retain the terminology used in the original reports. Although the two frameworks identify substantially overlapping populations, they are not strictly interchangeable because MASLD requires hepatic steatosis together with at least one cardiometabolic risk factor (6).

Notably, compared with MASLD in the general population, MASLD in PLWH involves several HIV-specific pathogenic mechanisms. These include persistent immune activation; HIV-associated immune injury to the intestinal mucosa and microbial translocation; metabolic dysregulation and disruption of the gut–liver axis; drug-specific metabolic effects and interactions associated with long-term ART; cellular stress induced by the HIV proteins transactivator of transcription (Tat) and gp120; accelerated cardiovascular risk; and increased visceral adiposity, together with distinct phenotypes such as lipodystrophy-associated and lean MASLD (1, 7–11). These HIV-specific factors support consideration of MASLD in PLWH as a distinct immunometabolic condition. Accordingly, this review integrates immune activation, gut–liver axis dysfunction, adipose tissue abnormalities, ART exposure, and changes in the hepatic immune microenvironment to explain disease progression.

For this narrative review, we searched PubMed for English-language studies published between 1996 and 2026. Search terms included “HIV”, “people living with HIV”, “PLWH”, “non-alcoholic fatty liver disease”, “NAFLD”, “metabolic dysfunction-associated steatotic liver disease”, “MASLD”, “metabolic dysfunction-associated steatohepatitis”, “MASH”, “hepatic steatosis”, “fibrosis”, “immune activation”, “inflammation”, “gut–liver axis”, “antiretroviral therapy”, and “diagnosis” and “treatment”. The terms were used individually and in relevant combinations. We included experimental and observational studies, clinical trials, systematic reviews, meta-analyses, editorials, and commentaries reporting evidence relevant to MASLD in PLWH. Evidence derived from the general MASLD population was also considered when HIV-specific data were unavailable or when such evidence provided important mechanistic, diagnostic, or therapeutic context.

## 2 Epidemiology

MASLD is highly prevalent among PLWH. Reported prevalence estimates range from 30% to 100% for NAFLD and from 20% to 89% for NASH, indicating a substantial disease burden in this population (12). These remarkably wide ranges likely reflect the aggregation of data from heterogeneous studies, with considerable differences in diagnostic modalities, indications for liver biopsy, histological definitions and thresholds, geographical regions, sex and age distributions, racial and ethnic backgrounds, metabolic risk profiles, viral hepatitis coinfections, and duration of ART exposure. Moreover, participants undergoing liver biopsy generally represent a clinically selected, high-risk population; therefore, studies using histology as the diagnostic reference standard may overestimate the prevalence of steatohepatitis and fibrosis.

A notable epidemiological feature of MASLD in PLWH is the relatively high prevalence of the lean phenotype. Screening strategies based solely on BMI or overt obesity may fail to identify a substantial proportion of PLWH at risk of MASLD, as a normal BMI does not exclude clinically significant hepatic steatosis or fibrosis. Current evidence suggests that approximately 45% of PLWH with NAFLD are not obese (13), while up to 24.2% of PLWH with a normal BMI meet the diagnostic criteria for NAFLD (14). Importantly, these individuals may still have more advanced liver fibrosis than their overweight or obese counterparts (15, 16). Accordingly, assessment in this population should extend beyond BMI and incorporate waist circumference, body fat distribution, metabolic abnormalities, ART history, and noninvasive fibrosis markers.

The reasons underlying the greater proportion of lean MASLD among PLWH than in the general population remain incompletely understood. Potential contributing factors include visceral adipose tissue accumulation despite a normal body weight, lipodystrophy, impaired subcutaneous adipose tissue storage capacity, ectopic lipid deposition, insulin resistance, mitochondrial dysfunction, chronic inflammation, cumulative ART exposure, sarcopenia, and genetic susceptibility.

## 3 Immunometabolic mechanisms

### 3.1 Direct effects of HIV infection

HIV infection promotes metabolic dysregulation and hepatic steatosis through direct viral effects and indirect mechanisms involving chronic immune activation and systemic inflammation (17, **Figure 1**). In individuals with biopsy-confirmed HIV-associated NAFLD, elevated circulating sCD14 and sCD163 levels were associated with adipose tissue dysfunction, indicated by an increased leptin-to-adiponectin ratio, as well as intrahepatic macrophage accumulation and liver fibrosis (18). Tesamorelin treatment reduced markers of monocyte and cytotoxic T-cell activation alongside changes in hepatic immune pathways, supporting a mechanistic link among metabolic dysfunction, systemic immune activation, and hepatic inflammation in PLWH (19).

**Figure 1:**
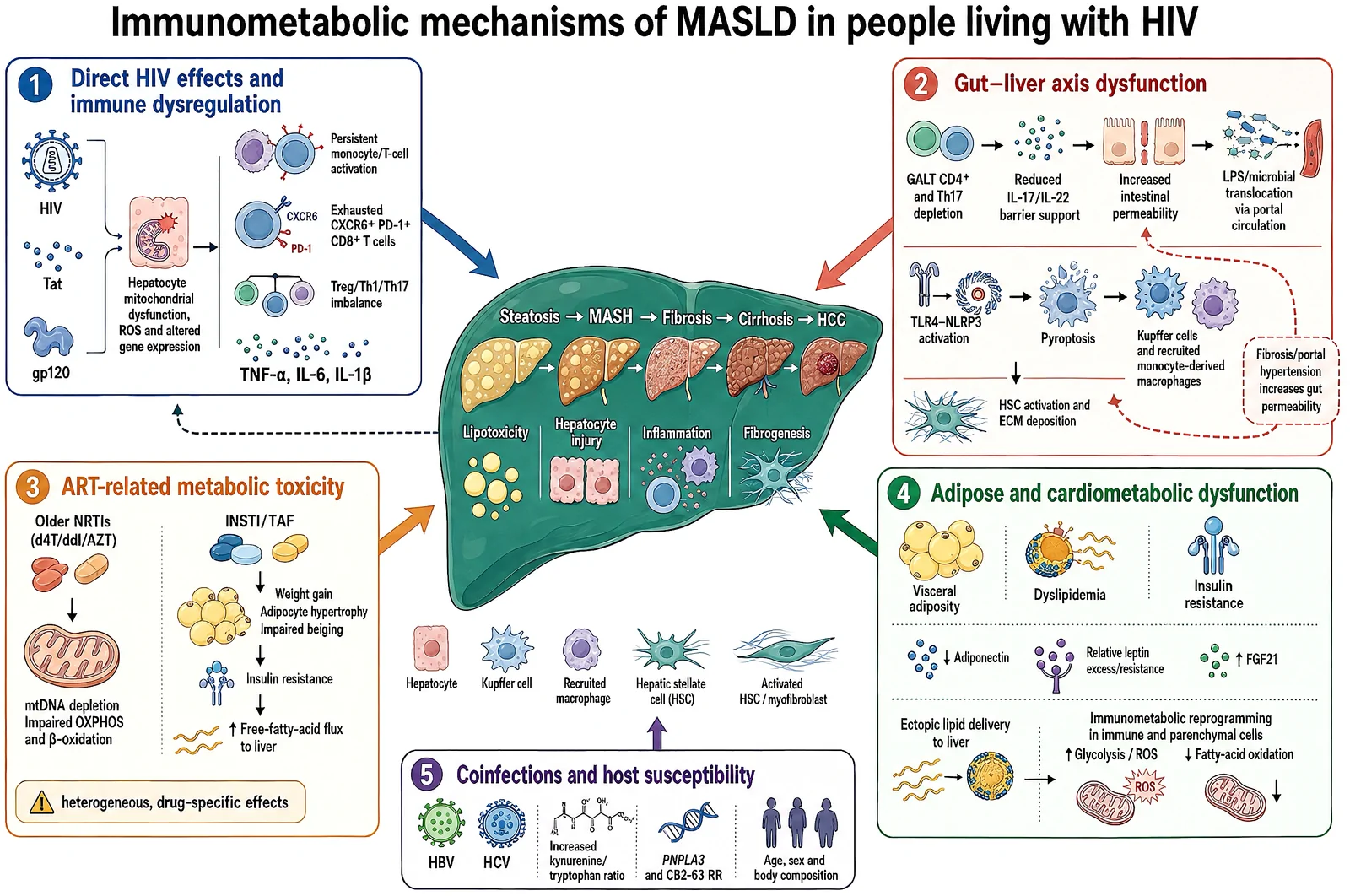
Immunometabolic mechanisms of MASLD in people living with HIV. Direct viral effects, persistent immune dysregulation, gut–liver axis disruption, ART-related metabolic toxicity, adipose/cardiometabolic dysfunction, coinfections, and host susceptibility converge on lipotoxicity, hepatocellular injury, inflammation, and fibrogenesis. GALT CD4+ and Th17 depletion promotes impaired barrier maintenance and microbial translocation. LPS-driven TLR4–NLRP3 signaling, pyroptosis, Kupffer-cell and monocyte-derived macrophage activation, and hepatic stellate-cell extracellular-matrix deposition reinforce disease progression. ART effects are drug-specific and heterogeneous; effective ART should not be discontinued solely because MASLD is present.

Regulatory T cells may have context-dependent roles in HIV-associated MASLD. Although Tregs can suppress generalized immune activation and tissue injury, they may also weaken HIV-specific effector responses and facilitate viral persistence (20). In MASH, an imbalance between Tregs and pro-inflammatory Th1, Th17, and cytotoxic CD8^+^ T cells may promote hepatic inflammation and fibrogenesis (21, 22). Because Treg abundance, phenotype, and function vary across the blood, adipose tissue, gut-associated lymphoid tissue, and liver, and direct studies in PLWH with MASLD remain limited, their effects cannot be considered uniformly protective or pathogenic.

The HIV Tat is essential for viral replication and contributes to HIV-related pathogenesis. Experimental studies in nonhepatic systems indicate that Tat can alter host signaling, oxidative stress, and mitochondrial function. Whether these effects directly promote hepatic steatosis in PLWH remains uncertain (23, 24). These effects may disrupt metabolic homeostasis, amplify inflammation, and promote tissue injury (25–27). In cultured human hepatic stellate cells, gp120 promoted chemotaxis and increased the expression of pro-inflammatory and profibrogenic mediators through CCR5-dependent signaling (28, 29). During chronic infection, these viral effects may sustain low-grade systemic inflammation, thereby promoting hepatic lipid accumulation, hepatocellular injury, and progression from steatosis to MASH, fibrosis, and cirrhosis (30).

The coexistence of HIV infection and metabolic dysfunction may generate chronic “metaflammation” and accelerate immunosenescence (31). Persistent monocyte and T-cell activation is closely associated with metabolic abnormalities, including insulin resistance (32, 33). Proteomic analysis of HIV-associated NAFLD further showed that F2–F3 fibrosis was associated with the upregulation of 20 plasma proteins involved in tissue repair and immune responses, particularly matrix metalloproteinase-2, insulin-like growth factor-binding protein 7, and collagen alpha 1 chain. These proteins were enriched in tumor necrosis factor-response and aminopeptidase pathways and were associated with visceral adiposity and impaired glucose tolerance (34).

Persistent antigenic stimulation and residual inflammation may also maintain dysfunctional or exhausted T cells despite suppressive ART (35, 36). T-cell exhaustion is characterized by increased expression of inhibitory receptors, including programmed cell death protein 1, T-cell immunoreceptor with immunoglobulin and ITIM domains, and T-cell immunoglobulin and mucin-domain-containing protein 3, together with impaired proliferation and altered cytokine production. Nevertheless, exhausted T cells may retain pathogenic activity. In experimental NASH, hepatic CXCR6^+^PD-1^+^ CD8^+^ T cells remained cytotoxic and promoted hepatocyte injury, suggesting that chronic antigenic stimulation and metabolic stress may jointly induce maladaptive T-cell responses (37).

### 3.2 Gut–liver axis dysregulation

HIV preferentially depletes CD4^+^ T cells within gut-associated lymphoid tissue (GALT), particularly Th17 cells, thereby disrupting IL-17- and IL-22-dependent maintenance of intestinal epithelial homeostasis, antimicrobial defense, and mucosal repair. Compared with women without HIV, women living with HIV exhibited lower circulating IL-17A levels but higher levels of IL-22 and sCD14 (38). A recent study further demonstrated that elevated kynurenine-to-tryptophan ratios and increased levels of sCD14 and sCD163 were associated with greater liver stiffness in both women with and without HIV. Moreover, biomarkers of microbial translocation mediated up to approximately 30% of the association between HIV infection and higher FibroScan–AST (FAST) scores (39). However, studies directly comparing the extent of intestinal Th17-cell depletion and GALT injury between individuals with HIV-associated MASLD and those with HIV-negative MASLD remain lacking.

Depletion of CD4^+^ T cells compromises intestinal barrier integrity and increases gut permeability, thereby facilitating the translocation of microbial products, such as lipopolysaccharide (LPS), into the portal circulation. LPS is a canonical ligand of Toll-like receptor 4 (TLR4), which is expressed by multiple hepatic cell populations, including hepatic stellate cells (HSCs), Kupffer cells (KCs), and hepatocytes (40).

In HSCs, TLR4 signaling promotes fibrogenesis through several mechanisms. For example, TLR4 activation suppresses the expression of bone morphogenetic protein and activin membrane-bound inhibitor, thereby enhancing transforming growth factor-β-mediated HSC activation and extracellular matrix deposition (41). Activated HSCs can also recruit and activate KCs through chemokine secretion, further reinforcing the profibrogenic hepatic microenvironment (41).

KCs are liver-resident macrophages that colonize the liver during embryonic development and constitute a critical component of the first-line innate immune defense. The liver also contains monocyte-derived macrophages, an infiltrating population recruited from circulating monocytes under inflammatory or infectious conditions. Despite their shared macrophage lineage, these populations may perform distinct functions during disease. An *in vivo* study of simian immunodeficiency virus infection in rhesus macaques suggested that recruited monocyte-derived macrophages may more closely reflect the magnitude of the infectious burden and microbial translocation, whereas KCs may contribute to specific metabolic and inflammatory alterations within the liver. Notably, KCs maintained high expression of the interferon-stimulated gene product MX1 throughout infection, consistent with a persistently activated, pro-inflammatory phenotype (42).

In hepatocytes, LPS exposure can induce lipotoxicity, apoptosis, and cellular stress responses. Injured hepatocytes subsequently release damage-associated molecular patterns, including high-mobility group box 1 and mitochondrial DNA, which further activate inflammatory cascades and perpetuate hepatic injury (43). In addition, activation of the LPS/TLR4 axis has been linked to NOD-like receptor family pyrin domain containing 3 inflammasome activation, pyroptosis, and increased release of interleukin-1β, thereby exacerbating hepatic inflammation and fibrosis (44, 45).

Hepatic macrophage responses in MASLD should not be interpreted according to a simple M1 or M2 dichotomy. Kupffer cells and recruited monocyte-derived macrophages comprise heterogeneous and highly plastic populations whose functions vary according to cellular origin, anatomical niche, disease stage, and local metabolic signals (46). During early metabolic injury, Kupffer cells sense lipotoxic hepatocyte-derived danger signals and gut-derived microbial products and produce inflammatory mediators, including TNF-α, IL-1β, and IL-6, thereby promoting monocyte recruitment and hepatic stellate-cell activation. With progression to NASH, resident Kupffer cells may partially lose their homeostatic identity or undergo cell death, while recruited monocytes differentiate into diverse inflammatory, lipid-associated, Kupffer-cell-like, and scar-associated macrophage populations (47). TREM2^+^CD9^+^ scar-associated macrophages accumulate within fibrotic niches and may contribute to stellate-cell activation and extracellular matrix remodeling (48). However, related macrophage programs may also facilitate lipid handling, efferocytosis, tissue repair, and fibrosis resolution under specific conditions. Thus, macrophage phenotypes represent a dynamic functional continuum rather than fixed pro-inflammatory or anti-inflammatory states. In PLWH, persistent microbial translocation and systemic immune activation may modify this macrophage landscape, although direct single-cell or spatial evidence in HIV-associated MASLD remains limited.

Alterations in gut microbial composition may also contribute to HIV-associated MASLD. Although HIV infection itself affects short-chain fatty acid-producing bacteria, MASLD appears to exert an even stronger influence on microbiome composition (49). Disease progression has been associated with distinct microbial signatures, including enrichment of genera such as *Ruminococcus* and *Holdemanella* and depletion of *Prevotella* and *Aliihoeflea* in patients with more advanced disease (50). Moreover, LPS has been reported to upregulate glutaminase 1, promoting hepatic ammonia accumulation and potentially accelerating progression toward MASH (51). HIV-associated mucosal injury and microbial translocation may therefore amplify hepatic inflammation and fibrogenesis.

### 3.3 Effects of ART

ART is essential for viral suppression, but the metabolic effects of individual agents may modify MASLD risk.

Older-generation nucleoside reverse transcriptase inhibitors (NRTIs), particularly the thymidine analogues stavudine and zidovudine and the purine analogue didanosine, can inhibit mitochondrial DNA polymerase γ. The resulting mitochondrial DNA depletion impairs oxidative phosphorylation and fatty acid β-oxidation, thereby promoting insulin resistance, hepatic lipid accumulation, and steatosis. Clinically, this toxicity ranges from asymptomatic aminotransferase elevations to severe lactic acidosis and macrovesicular steatosis (52–54). In a cohort of 671 individuals with HIV/HCV coinfection, exposure to didanosine for more than 5 months was independently associated with advanced fibrosis (odds ratio, 1.7; 95% confidence interval, 1.0–2.8; P = 0.04) (55). However, this association was observed in a coinfected population and should not be directly extrapolated to contemporary ART-treated PLWH with MASLD.

Contemporary NRTIs have substantially less mitochondrial toxicity, although their metabolic effects may differ. Tenofovir alafenamide (TAF) exposure has been associated with weight gain, metabolic abnormalities, and the development or progression of hepatic steatosis, whereas tenofovir disoproxil fumarate (TDF) has been associated with a comparatively more favorable weight and lipid profile (56). Interpretation is complicated because TAF is frequently combined with an integrase strand transfer inhibitor (INSTI) and because changes observed after switching from TDF to TAF may reflect both the introduction of TAF and withdrawal of the weight- and lipid-suppressive effects of TDF.

INSTIs appear to have limited intrinsic hepatotoxicity and may affect hepatic steatosis primarily through changes in body weight, adipose tissue function, and insulin sensitivity (57). Among women with HIV, INSTI initiation was associated with 3.7-fold higher odds of hepatic steatosis, defined by a controlled attenuation parameter (CAP) ≥248 dB/m, during the first year of treatment (58). The association was largely confined to the first year after initiation, suggesting an early metabolic effect rather than a sustained increase in progressive liver disease risk.

Mechanistic evidence supports this interpretation. PLWH who gained more than 10% of body weight within 18 months of initiating an INSTI-based regimen exhibited accumulation of medium-chain acylcarnitines, impaired mitochondrial fatty acid oxidation, and increased insulin resistance (59). Experimental studies further suggest that dolutegravir and raltegravir promote adipocyte differentiation, hypertrophy, extracellular matrix deposition, and insulin resistance, whereas dolutegravir and bictegravir may inhibit adipose tissue beiging (60, 61). These alterations may reduce the capacity of adipose tissue to store excess lipid safely, increase free fatty acid delivery to the liver, and promote intrahepatic triglyceride accumulation.

Adipose tissue dysfunction in PLWH also involves altered adipokine secretion. Reduced adiponectin is associated with impaired insulin sensitivity, dyslipidemia, and increased liver fat, whereas the effects of leptin vary according to peripheral lipoatrophy and visceral adiposity (62). Relative leptin excess or leptin resistance may promote inflammatory and fibrogenic signaling (63), while elevated FGF21 may reflect a compensatory response to metabolic stress (64). Therefore, the leptin-to-adiponectin ratio is a useful marker of adipose dysfunction but does not fully represent the heterogeneous adipokine profiles of PLWH (18). Direct evidence linking these alterations to MASLD progression remains limited.

Nevertheless, findings across INSTI studies remain inconsistent. Differences in sex, baseline adiposity and metabolic risk, concomitant TAF exposure, previous TDF use, duration of follow-up, and methods used to define steatosis or fibrosis may partly explain this heterogeneity. Current evidence therefore supports metabolic monitoring after ART initiation or modification but does not establish a uniform direct hepatotoxic effect of INSTIs.

### 3.4 Viral coinfections

In many PLWH, the hepatic burden imposed by HIV and ART is further compounded by viral coinfections. Because of shared routes of transmission, coinfection with HBV or HCV is common and can substantially aggravate liver disease severity. In people with HIV/HBV coinfection, the prevalence of fatty liver disease has been reported to be approximately 30%, with around 10% progressing to steatohepatitis (65).

In recent years, tryptophan catabolism has emerged as a potentially important biological pathway linking alterations in the gut microbiota, immune activation, and liver fibrosis. One study demonstrated that the plasma kynurenine-to-tryptophan ratio was significantly elevated among PLWH. The median ratio was 0.038 in those with HIV monoinfection and 0.056 in those with HIV/HCV coinfection, compared with 0.0312 in women without either infection (66). This stepwise increase suggests that HIV infection itself may enhance tryptophan catabolism, with HCV coinfection further amplifying this effect. After adjustment for sociodemographic, lifestyle, and metabolic factors, a higher kynurenine-to-tryptophan ratio remained independently associated with an elevated f Four imaging modalities are commonly used to detect and quantify hepatic steatosisibrosis-4 (FIB-4) index. Each twofold increase in the kynurenine-to-tryptophan ratio was associated with a 27% higher FIB-4 index.

### 3.5 Host genetic susceptibility

Host genetic background may partly explain why MASLD susceptibility and disease severity vary substantially among PLWH exposed to similar viral and metabolic stressors. Genetic predisposition appears to modulate the hepatic response to chronic inflammation, metabolic dysfunction, and immune dysregulation.

In HIV/HCV-coinfected individuals, the CB2-63 RR variant has been independently associated with more severe hepatic necroinflammation. Associations with steatosis and fibrosis have also been reported, but independent effects on these outcomes remain uncertain (67, 68). In addition, polymorphisms in the patatin-like phospholipase domain-containing protein 3 gene have been strongly associated with an increased risk of hepatic steatosis in HIV-infected populations.

Although the precise mechanistic roles of these genetic variants remain incompletely understood, current evidence suggests that host genetic background may influence baseline susceptibility to chronic liver injury and modulate disease severity in the presence of metabolic stress and HIV-related immune dysregulation (69).

### 3.6 Immunometabolic reprogramming

Immunometabolic reprogramming may represent an important mechanistic link between persistent immune activation and MASLD progression in PLWH. A human serum metabolomic study demonstrated increased glycolytic activity, lactate production, and pentose phosphate pathway flux in PLWH, accompanied by impaired long-chain fatty acid β-oxidation. Notably, some of these metabolic abnormalities persisted despite virologically suppressive ART (70).

Enhanced glycolytic flux can support immune-cell activation and pro-inflammatory cytokine production, whereas defective fatty acid oxidation and mitochondrial dysfunction may promote lipid accumulation, disruption of the mitochondrial electron transport chain, and generation of reactive oxygen species. Reactive oxygen species and lipotoxic intermediates can, in turn, activate nuclear factor-κB signaling and inflammasome-dependent inflammatory pathways. Conversely, pro-inflammatory cytokines released by activated T cells, monocytes, and hepatic macrophages can impair insulin signaling, promote adipose tissue lipolysis, increase the delivery of free fatty acids to the liver, and further compromise mitochondrial lipid oxidation (71).

Metabolic dysfunction and immune activation may form a self-reinforcing cycle in PLWH (30). Persistent inflammation impairs insulin signaling and mitochondrial lipid oxidation, whereas lipid accumulation and oxidative stress further activate inflammatory pathways. This interaction may promote steatosis and fibrosis even in individuals without obesity (14, 72), providing a mechanistic basis for lean MASLD (14). Because insulin resistance, atherogenic dyslipidemia, monocyte activation, oxidative stress, and endothelial dysfunction also promote cardiovascular disease (73), hepatic steatosis and fibrosis may identify broader systemic immunometabolic risk even in young or normal-weight PLWH.

## 4 Diagnosis

MASLD is an important comorbidity among PLWH, and its diagnostic pathway differs from that applied to HIV infection alone or MASLD without HIV. In addition, differences exist in the diagnostic methods and their applicability, and relevant references are provided for further consideration (**Table 1**).

**Table 1:** Diagnostic methods for MASLD in people living with HIV.

Method	What it assesses	Advantages	Diagnostic performance	Evidence specificity
Etiological exclusion and cardiometabolic assessment	History; alcohol exposure; HBV/HCV testing; medication review; liver biochemistry; anthropometry; glucose and lipid assessment; consider HOMA-IR/OGTT when suspicion remains high.	Identifies competing or coexisting liver injury and establishes the MASLD cardiometabolic criterion; essential in PLWH with multifactorial disease.	No single diagnostic cutoff. MASLD requires hepatic steatosis plus ≥1 cardiometabolic risk factor after exclusion of alternative causes.	Clinical framework
FIB-4	Age, AST, ALT, and platelet count.	Low cost, widely available, reproducible; useful for first-line exclusion of advanced fibrosis.	<1.3: low risk; 1.3–2.67: indeterminate; ≥2.67: high risk.	Originally developed in HIV/HCV; MASLD cutoffs largely guideline-derived
APRI	AST relative to its upper limit of normal and platelet count.	Simple, inexpensive adjunct when FIB-4 is unavailable or requires corroboration.	<0.5: low likelihood of significant fibrosis; ≥0.5: cannot exclude significant fibrosis and should prompt TE.	Threshold extrapolated from general NAFLD/MASLD populations.
Routine liver biochemical tests	ALT, AST, ALP, total/direct bilirubin.	Accessible; detects hepatocellular injury, cholestasis, or impaired excretory function; supports differential diagnosis and follow-up.	No MASLD-specific diagnostic threshold in the review.	Supportive, not confirmatory.
Lipid parameters	TG and HDL-C, including trends after ART initiation.	Reflect the characteristic atherogenic metabolic phenotype and identify cardiometabolic risk.	Independent predictors reported in PLWH: baseline TG ≥1.7 mmol/L, persistent TG elevation after ART initiation, and HDL-C <40 mg/dL.	Associations reported in PLWH.
Fatty liver index (FLI)	BMI, waist circumference, TG, and GGT.	Non-invasive, inexpensive steatosis screen; better calibrated than HSI in the cited HIV cohort.	FLI ≥60: C-index 0.85 (95% CI 0.80–0.89), with satisfactory calibration in an HIV cohort.	Direct HIV-cohort evidence.
Hepatic steatosis index (HSI)	ALT/AST ratio, BMI, sex, and diabetes status.	Simple serum-based steatosis screen.	HSI ≥36: C-index 0.78 (95% CI 0.73–0.83).	Direct HIV-cohort evaluation.
Conventional ultrasonography	Qualitative echogenic features of hepatic steatosis.	Widely available, inexpensive, no radiation.	No numeric performance estimate or threshold provided in the review.	General clinical evidence.
CAP with VCTE	CAP quantifies ultrasound attenuation; same-session LSM estimates fibrosis.	Rapid, non-invasive, point-of-care; simultaneously assesses steatosis and fibrosis; PLWH-specific CAP cutoff available.	CAP ≥285 dB/m vs MRI-PDFF ≥5%: sensitivity ≈73%, specificity 78.6%, PPV 93.2% in HIV-associated NAFLD.	CAP cutoff derived specifically in PLWH.
LSM by VCTE	Liver stiffness in kPa.	Fast, repeatable and clinically scalable fibrosis stratification.	<8 kPa: low likelihood of significant fibrosis; ≥8 kPa: increased risk; >11 kPa: high risk of advanced fibrosis.	Applied in PLWH cohorts but thresholds largely derived from MASLD guidance.
MRI-PDFF	Quantitative proton-density fat fraction across the liver.	Non-invasive reference standard for liver-fat quantification; highly sensitive, reproducible, and useful for treatment response.	≥5% defines hepatic steatosis; used as the reference in the PLWH CAP study.	Threshold not originally HIV-specific but repeatedly evaluated in PLWH.
Magnetic resonance elastography (MRE)	MRI-based quantitative liver stiffness.	High accuracy for fibrosis; samples a larger liver volume; useful when VCTE fails or is unreliable; may avoid biopsy.	General-population thresholds: <2.5–3.0 kPa suggests no clinically significant fibrosis; ≥3.6–3.7 kPa suggests increased likelihood of advanced fibrosis.	Thresholds extrapolated from general populations.
Liver biopsy	Histological assessment of steatosis, ballooning, inflammation, MASH, and fibrosis stage.	Reference standard; distinguishes isolated steatosis from MASH; evaluates competing diagnoses and accurately stages fibrosis.	Histology-based reference; no single numeric cutoff summarized.	Definitive diagnostic standard.

Diagnostic performance and cutoffs are reported exactly as summarized in the review. “PLWH-specific” indicates direct evaluation in cohorts of people living with HIV. Other thresholds should be interpreted as extrapolated unless stated otherwise.

### 4.1 Exclusion of alternative etiologies and assessment of metabolic risk

The diagnosis of MASLD requires the exclusion of alternative causes of hepatic steatosis. This step is particularly important in PLWH because multiple hepatic insults frequently coexist in this population. During the initial evaluation, the presence of hepatic steatosis should be confirmed, while viral hepatitis coinfection, alcohol-associated liver disease, drug-induced liver injury, monogenic disorders, and other causes of liver injury should be systematically excluded (1). Notably, HIV infection itself has been recognized as a potential contributor to hepatic steatosis.

Within the diagnostic pathway for MASLD, individuals without overt cardiometabolic risk factors but with a strong clinical suspicion of metabolic dysfunction may be considered to have possible early-stage MASLD and should undergo further metabolic evaluation, including the homeostatic model assessment for insulin resistance and an oral glucose tolerance test. HIV-specific contributors, including the direct effects of viral proteins and the metabolic consequences of ART, should also be considered.

By definition, MASLD requires the presence of hepatic steatosis together with at least one cardiometabolic risk factor. In PLWH, the assessment should systematically encompass obesity and body fat distribution, type 2 diabetes and insulin resistance, individual components of the metabolic syndrome, and anthropometric parameters, including waist circumference, hip circumference, and the waist-to-hip ratio.

### 4.2 Initial screening using FIB-4 as the primary tool and APRI as an adjunct

The FIB-4 index was originally developed to facilitate the assessment of liver fibrosis in individuals with HIV/HCV coinfection and is now widely used for noninvasive fibrosis risk stratification, particularly in patients with MASLD (74). In the general population, a FIB-4 value <1.3 indicates a low risk and is commonly used to exclude advanced fibrosis; values between 1.3 and 2.67 are considered indeterminate; and values ≥2.67 indicate a high risk of advanced liver fibrosis. However, a study conducted among PLWH demonstrated that the diagnostic performance of FIB-4 was particularly limited in those with concomitant MASLD. Although a FIB-4 value <1.3 retained a relatively high negative predictive value, its sensitivity was suboptimal. Conversely, a FIB-4 value ≥2.67 showed high specificity but a limited positive predictive value. The area under the receiver operating characteristic curve was only 0.60 in participants with MASLD, compared with 0.76 in those without MASLD (75). Therefore, FIB-4 should be used primarily as an initial risk-stratification tool. In PLWH with a high metabolic risk burden, further assessment using transient elastography (TE) should be considered even when FIB-4 is <1.3.

The APRI, calculated from routinely measured aspartate aminotransferase (AST) and platelet values, may serve as an adjunctive first-line tool for fibrosis risk stratification. An APRI value <0.5 indicates a low likelihood of significant liver fibrosis, and TE may not be immediately required. Nevertheless, TE should still be considered in PLWH with obesity, hypertriglyceridemia, diabetes, or other prominent metabolic risk factors, even when APRI is <0.5. An APRI value ≥0.5 does not exclude significant liver fibrosis and should prompt further evaluation with TE. An APRI threshold <0.5 has been evaluated in two-tier fibrosis pathways involving PLWH and may reduce unnecessary TE referrals. However, these studies primarily used TE rather than liver histology as the reference standard, and the threshold has not been fully validated in biopsy-defined HIV-associated MASLD (76).

### 4.3 Serological diagnosis

Abnormal lipid metabolism is closely associated with the development, progression, and severity of steatotic liver disease in PLWH, among whom atherogenic dyslipidemia represents a characteristic metabolic phenotype. Several lipid-related parameters have been identified as independent predictors of MASLD in PLWH, including a baseline triglyceride (TG) level ≥1.7 mmol/L, persistently elevated TG levels after ART initiation, and reduced high-density lipoprotein cholesterol levels (<40 mg/dL) (77, 78).

The fatty liver index (FLI) is a noninvasive scoring system used to identify hepatic steatosis. In a study of an HIV cohort, an FLI threshold ≥60 showed good diagnostic performance for hepatic steatosis, with a C-index of 0.85 (95% confidence interval [CI], 0.80–0.89) and satisfactory calibration (79). The hepatic steatosis index (HSI) can also be used to detect hepatic steatosis in PLWH. At a threshold ≥36, the HSI achieved a C-index of 0.78 (95% CI, 0.73–0.83). However, the HSI tended to overestimate the probability of steatosis in PLWH, whereas the FLI demonstrated superior diagnostic performance and greater clinical utility. Furthermore, the estimated prevalence of MASLD based on these indices tends to increase with the duration of ART exposure, potentially reflecting the cumulative metabolic effects of long-term treatment (77).

Routine liver biochemical tests support the assessment of hepatocellular injury, cholestasis, and hepatic excretory function but cannot exclude steatosis, MASH, or fibrosis when values are normal. Persistent abnormalities should prompt evaluation for viral hepatitis, drug-induced liver injury, alcohol-related liver disease, and other coexisting liver disorders.

### 4.4 Imaging assessment

Imaging is central to the noninvasive assessment of MASLD in PLWH and is primarily used to evaluate hepatic steatosis and fibrosis. Several imaging modalities are used to assess hepatic steatosis and fibrosis.

First, vibration-controlled transient elastography (VCTE) combined with the CAP enables the simultaneous assessment of hepatic steatosis and liver stiffness during a single FibroScan examination. CAP is used to estimate hepatic fat content, whereas the liver stiffness measurement (LSM) obtained using VCTE is used to assess fibrosis. In a study using magnetic resonance imaging–proton density fat fraction (MRI-PDFF) ≥5% as the reference standard for HIV-associated NAFLD, a CAP threshold of ≥285 dB/m was identified as the optimal cutoff for detecting hepatic steatosis. At this threshold, CAP had a sensitivity of approximately 73%, a specificity of 78.6%, and a positive predictive value of 93.2%, providing a threshold derived specifically from PLWH (80).

LSM values may be interpreted concurrently to stratify fibrosis risk. In contemporary HIV cohorts, LSM ≥8 kPa has been used to identify clinically significant fibrosis, whereas thresholds of ≥11–12 kPa have been used for advanced fibrosis. These cutoffs remain insufficiently validated against liver histology in HIV-associated MASLD (72, 81).

MRI-PDFF is widely regarded as the noninvasive reference standard for quantifying hepatic fat content because of its high sensitivity and reproducibility (82). MRI-PDFF should be considered when CAP and LSM findings are discordant or when the patient has a high metabolic risk burden. An MRI-PDFF value ≥5% is commonly used to define the presence of hepatic steatosis. Although this threshold was not originally developed specifically for PLWH, it has been repeatedly applied and extensively evaluated in studies involving PLWH (83).

Magnetic resonance elastography (MRE) may be useful when serum-based indices and VCTE findings are discordant, VCTE is unsuccessful or unreliable, obesity or body habitus limits measurement accuracy, advanced fibrosis remains clinically suspected despite inconclusive tests, or confirmation may avoid liver biopsy (84).

Conventional ultrasonography remains a widely available and cost-effective first-line imaging modality. It identifies hepatic steatosis on the basis of characteristic echogenic features but has limited sensitivity for mild steatosis, and its diagnostic performance is operator dependent (85).

### 4.5 Definitive diagnosis: liver biopsy

Liver biopsy remains the reference standard for distinguishing isolated steatosis from MASH and for accurately staging liver fibrosis; however, its invasive nature precludes its use as a population-wide screening tool. Liver biopsy may be considered when FIB-4 or APRI findings are markedly discordant with TE results, LSM or MRE findings persistently indicate advanced fibrosis, MASH is suspected and histological confirmation would influence treatment decisions or eligibility for clinical trials, or liver enzyme elevations persist without an identifiable cause. Biopsy may also be required when the differential diagnosis includes drug-induced liver injury, autoimmune liver disease, viral hepatitis, alcohol-associated liver disease, or other hepatic disorders.

## 5 Management

### 5.1 Foundational management

Lifestyle modification remains the cornerstone of MASLD management and has also demonstrated benefits among PLWH. MASLD is an important comorbidity among PLWH, and its diagnostic pathway differs from that applied to HIV infection alone or MASLD without HIV (**Table 2**). Recommended interventions include adopting a Mediterranean-style diet or an individualized energy-restricted dietary pattern; reducing the consumption of sugar-sweetened beverages, fructose, refined carbohydrates, and highly processed foods; engaging in at least 150 min of moderate-intensity aerobic exercise per week, supplemented by resistance training two to three times weekly; limiting alcohol consumption; reducing sedentary behavior; improving sleep quality; and promoting smoking cessation (86).

**Table 2:** Management options and strength of evidence for HIV-associated MASLD.

Intervention	Target population	Benefits	Strength of evidence	Clinical position
Lifestyle and weight management	Mediterranean-style or individualized energy-restricted diet; reduce fructose/refined/ultraprocessed foods	Improves weight, visceral adiposity and cardiometabolic risk	Moderate overall	Established standard of care.
Cardiometabolic risk control	Treat diabetes, hypertension, dyslipidemia, obesity and visceral adiposity	Reduces major extrahepatic risk and may improve metabolic drivers of MASLD	High for standard cardiometabolic/CV indications	Established standard of care.
Maintain suppression and optimize ART	Continue effective ART	Preserves HIV control while reducing avoidable drug-related metabolic injury	High for continued virological suppression	Established HIV care with individualized MASLD modification.
GLP-1 receptor agonists	Established obesity or type 2 diabetes indications	Weight and visceral-fat reduction; improved insulin resistance/TG; cardiovascular benefit in selected patients; potential liver-fat reduction	High for approved metabolic indications	Prefer when standard obesity/diabetes indication coexists
Semaglutide	Use according to established indications	General phase 3 data show MASH-resolution and fibrosis-related histological benefit	High for general-population approved indication	Promising in PLWH with obesity/T2DM or eligible MASH
SGLT2 inhibitors	When T2DM, CKD or heart failure provides a standard indication	May reduce liver fat and aminotransferases; established cardiorenal benefit in appropriate populations	High for standard cardiorenal indications	Do not prescribe solely for MASLD; useful when standard indications coexist.
Tesamorelin	FDA-approved GHRH analogue for excess visceral abdominal fat in adults with HIV	Reduced visceral adipose tissue, hepatic fat fraction and trunk-to-limb fat ratio	Moderate PLWH-specific evidence for visceral/hepatic fat reduction	Most directly supported HIV-specific pharmacologic option for abdominal fat accumulation with hepatic steatosis.
Resmetirom	Approved with diet/exercise for noncirrhotic MASH with moderate-to-advanced fibrosis	Reduces hepatic fat, inflammation/fibrosis and circulating lipids in general-population trials	High for eligible general-population MASH	Potential option only after specialist confirmation of eligibility and careful interaction/safety review; evidence cannot be assumed equivalent in PLWH.
Rilpivirine	Preclinical/translational proposal	Possible attenuation of hepatic injury and fibrosis	Mechanistic/translational evidence only	Not recommended as MASLD-directed ART selection.
Microbiome-directed interventions	Probiotics, prebiotics, fecal microbiota transplantation and gut-barrier repair strategies	Target dysbiosis, microbial translocation and gut–liver immune signaling	Investigational.	Clinical trials only
Immunomodulatory approaches	Potential targeting of monocyte/macrophage activation, inflammasome pathways, T-cell exhaustion/checkpoints or inflammatory signaling	Mechanistically aligned with HIV-associated immune activation and fibrogenesis	Investigational.	Mechanistic studies and carefully monitored trials only
Integrated multidisciplinary follow-up	Coordinate HIV, hepatology, metabolic and cardiovascular care	Balances virological efficacy, liver risk, CVD prevention, polypharmacy and drug interactions	Consensus/clinical-practice evidence; direct outcome trials needed	Recommended care model

Evidence grades are pragmatic categories based on the population specificity and study design described in the review, not a formal GRADE assessment.

Weight management is another essential component of care. A weight loss target of approximately 7%–10% is recommended for PLWH who are overweight or obese, whereas a more modest target of approximately 3%–5% may be appropriate for individuals with normal body weight or lean MASLD (86). Particular attention should be paid to preserving skeletal muscle mass and preventing sarcopenia. In a previous study, weight loss of ≥10% was associated with a reduction in the NAFLD activity score in all participants, resolution of NASH in 90%, and regression of fibrosis in 45%, suggesting that weight loss of this magnitude is more likely to improve NASH and hepatic fibrosis (87). However, these findings were derived from the general MASLD population and should therefore be extrapolated cautiously to PLWH with MASLD.

The management of MASLD in PLWH should also include comprehensive control of cardiometabolic comorbidities, including diabetes, hypertension, dyslipidemia, obesity, and visceral adiposity (1). Cardiovascular risk should be systematically assessed, and statin therapy should be prescribed according to established indications, with consideration of potential drug–drug interactions with ART (88).

### 5.2 Optimization of ART regimens

Effective ART should be continued, and the identification of MASLD or abnormal liver biochemical findings alone should not prompt treatment discontinuation. Maintenance of virological suppression remains the primary objective of HIV treatment (1).

ART-related metabolic risk should nevertheless be reviewed as part of the management of MASLD. Older NRTIs with established mitochondrial toxicity and lipodystrophy risk, particularly stavudine and didanosine, should be avoided when clinically feasible. Following initiation of an INSTI-containing regimen or a switch from TDF to TAF, changes in body weight, waist circumference, glucose metabolism, and lipid profiles should be monitored, particularly in patients with pre-existing cardiometabolic risk (53, 89, 90).

However, current evidence does not support routine switching of an otherwise effective INSTI- or TAF-containing regimen solely because MASLD is present. Any modification should be individualized according to the temporal relationship between ART exposure and metabolic deterioration, the severity of MASLD, virological efficacy, resistance history, previous treatment exposure, drug–drug interactions, and renal and bone safety (53).

Certain antiretroviral agents may also have hepatic effects independent of viral suppression. Translational evidence suggests that rilpivirine may attenuate hepatic injury and fibrosis through activation of signal transducer and activator of transcription 1 signaling in non-parenchymal liver cells (91). However, these findings are insufficient to recommend preferential use of rilpivirine specifically for the treatment of MASLD.

### 5.3 GLP-1 receptor agonists and SGLT2 inhibitors

Several pharmacological therapies have demonstrated metabolic or hepatic benefits in the general population with MASLD, obesity, or diabetes mellitus. However, evidence specifically supporting their use in PLWH remains limited. These therapies include glucagon-like peptide-1 receptor agonists (GLP-1 RAs) and sodium–glucose cotransporter 2 (SGLT2) inhibitors.

GLP-1 RAs, including semaglutide, liraglutide, and dulaglutide, effectively reduce body weight and visceral adiposity in individuals who are overweight or obese. They may also improve insulin resistance, reduce TG levels, and confer cardiovascular benefits in appropriately selected patients (92). Accordingly, GLP-1 RAs may be preferentially considered in PLWH who have established indications such as obesity or type 2 diabetes mellitus. Potential gastrointestinal adverse effects and the risk of gallbladder disease should be considered, while rapid weight loss should be carefully monitored to minimize unintended consequences, including excessive loss of lean body mass.

SGLT2 inhibitors, such as empagliflozin and dapagliflozin, may be considered for PLWH with established indications, including type 2 diabetes mellitus, chronic kidney disease, or heart failure. Studies have suggested that these agents may reduce hepatic fat content and improve aminotransferase levels (93, 94). However, evidence in PLWH is currently derived from small observational studies, and dedicated randomized controlled trials using MRI-PDFF, histological resolution of MASH, or fibrosis improvement as primary endpoints remain lacking (95). Therefore, SGLT2 inhibitors should not be prescribed to PLWH solely for the treatment of MASLD. Clinicians should also consider the risks of genital mycotic infections, volume depletion, and the rare occurrence of euglycemic diabetic ketoacidosis (96).

### 5.4 Tesamorelin and HIV-specific evidence for semaglutide

Tesamorelin is a growth hormone–releasing hormone analog approved by the FDA for reducing excess visceral adipose tissue in adults with HIV-associated abdominal fat accumulation. A randomized, double-blind trial involving PLWH with MASLD further demonstrated that 12 months of tesamorelin treatment significantly reduced visceral adipose tissue area and hepatic fat fraction and improved the trunk-to-limb fat ratio (97, 98). However, evidence supporting its ability to reverse established hepatic fibrosis remains insufficient.

Wegovy received accelerated approval from the FDA for adults with noncirrhotic MASH and F2–F3 fibrosis. This approval was based on interim phase 3 data showing that semaglutide improved MASH resolution and fibrosis-related histological outcomes compared with placebo (99). However, whether these improvements reduce cirrhosis, hepatic decompensation, transplantation, or mortality remains under investigation. Evidence in PLWH remains limited. In the 24-week, single-arm SLIM LIVER study, semaglutide reduced liver fat by approximately 31% in 49 virologically suppressed PLWH with MASLD, and 29% achieved an MRI-PDFF of ≤5% (100). However, the study lacked a placebo group and histological assessment. Therefore, the efficacy of semaglutide for MASH resolution or fibrosis regression in PLWH remains unproven, and the general-population approval of Wegovy should not be directly extrapolated to this population.

### 5.5 Resmetirom

Resmetirom is the first oral therapy approved by the FDA for the treatment of noncirrhotic MASH with moderate-to-advanced hepatic fibrosis and is indicated for use in conjunction with dietary modification and exercise. As a liver-directed, selective thyroid hormone receptor-β agonist, resmetirom enhances hepatic lipid metabolism and clearance, reduces hepatic fat and circulating atherogenic lipids and improved histological endpoints of MASH resolution and fibrosis in the MAESTRO-NASH trial (101, 102). However, PLWH were not included in the pivotal MAESTRO clinical trials. Consequently, the efficacy, safety, and pharmacokinetic profile of resmetirom in PLWH receiving long-term ART remain insufficiently characterized.

### 5.6 Integrated management

Management should integrate HIV control, cardiometabolic risk reduction, and fibrosis-based liver surveillance. Follow-up intensity should be determined by fibrosis stage and metabolic risk, with hepatology referral for indeterminate, discordant, or high-risk findings. Pharmacotherapy should be selected according to established indications and the strength of HIV-specific evidence. ART modification should not compromise virological suppression, and potential drug–drug interactions should be reviewed regularly.

## 6 Future directions and emerging research priorities

Despite recent advances, major knowledge gaps remain in HIV-associated MASLD, including its incompletely defined natural history, heterogeneous clinical phenotypes, suboptimal risk-stratification tools, and limited HIV-specific therapeutic evidence. Longitudinal prospective cohorts are needed to characterize progression from steatosis to fibrosis, cirrhosis, and HCC and to identify predictors of hepatic and extrahepatic outcomes (103). These studies should include populations that remain underrepresented in current research and should examine the modifying effects of sex, age, menopausal status, ethnicity, and geographic setting (104).

The effects of viral coinfections also require further investigation, particularly the persistence of metabolic and lipid abnormalities after HCV cure in PLWH (105). Risk-prediction models should integrate metabolic, inflammatory, virological, and imaging variables and should be prospectively validated against clinically relevant liver outcomes. Emerging biomarkers, including soluble CD163, require validation beyond cross-sectional associations before they can be incorporated into routine clinical practice (106).

Therapeutic research should prioritize adequately powered randomized trials specifically enrolling PLWH. Such studies should evaluate histological resolution of MASH, fibrosis regression, cardiovascular outcomes, safety, drug–drug interactions, and durability of treatment response rather than relying solely on changes in body weight, aminotransferase levels, or hepatic fat content. The inconclusive findings from early studies of maraviroc and metformin illustrate the need for more rigorous trial designs and clinically meaningful endpoints (107).

Future studies should also determine whether responses to GLP-1 receptor agonists, tesamorelin, resmetirom, and other metabolic therapies differ according to ART regimen, baseline fibrosis stage, sex, adipose tissue distribution, or inflammatory phenotype. Immunomodulatory and microbiome-directed interventions require mechanistic studies followed by carefully designed clinical trials before they can be considered for routine management. Although statins have an established role in cardiovascular prevention among PLWH, whether they also modify MASLD progression remains uncertain (88, 108, 109).

Emerging data also indicate that immune checkpoint inhibitors can be administered to selected PLWH with cancer without an apparent excess of unexpected immune-related toxicity, particularly when HIV is virologically suppressed and care is coordinated across oncology and HIV services (110, 111). However, these oncology data should not be interpreted as evidence that checkpoint blockade is an effective or appropriate treatment for HIV-associated MASLD. Rather, they support the feasibility of carefully designed mechanistic studies examining immune exhaustion and checkpoint pathways in PLWH, with close monitoring of hepatic toxicity, immune status, and HIV control.

Finally, future research should assess whether integrated multidisciplinary care improves hepatic, cardiovascular, and patient-reported outcomes. Future models should determine whether inflammatory biomarkers improve fibrosis prediction beyond clinical variables and imaging.

## 7 Conclusion

MASLD in PLWH reflects the interaction of conventional metabolic risk factors with HIV-related immune activation, gut–liver axis disruption, altered adipose function, and ART-associated metabolic effects. These mechanisms may contribute to clinically significant disease even in individuals without obesity. Management should combine cardiometabolic risk control, continued effective ART, and fibrosis-based risk stratification. Although several metabolic therapies reduce body weight or hepatic fat, HIV-specific evidence for MASH resolution, fibrosis regression, and long-term clinical outcomes remains limited.
